# Whole exome sequencing for determination of tumor mutation load in liquid biopsy from advanced cancer patients

**DOI:** 10.1371/journal.pone.0188174

**Published:** 2017-11-21

**Authors:** Florence Koeppel, Steven Blanchard, Cécile Jovelet, Bérengère Genin, Charles Marcaillou, Emmanuel Martin, Etienne Rouleau, Eric Solary, Jean-Charles Soria, Fabrice André, Ludovic Lacroix

**Affiliations:** 1 Genomic Platform–Molecular Biopathology Unit and Biological Resource Center, AMMICA, INSERM US23/CNRS UMS3655, Gustave Roussy, Villejuif, France; 2 IntegraGen SA, Genopole CAMPUS 1 bat, G8, EVRY, Paris, France; 3 Department of Medical Biology and Pathology, Gustave Roussy, Villejuif, France; 4 INSERM U1170, Gustave Roussy, Faculté de Médecine Paris-Sud, Université Paris-Saclay, Villejuif, France; 5 Department of Hematology, Gustave Roussy, Villejuif, France; 6 Drug Development Department (DITEP), Gustave Roussy, and Université Paris-Sud, Villejuif, France; 7 Department of Medical Oncology, Gustave Roussy, Villejuif, France; 8 INSERM U981, Gustave Roussy, Université Paris XI, Villejuif, France; Carolina Urologic Research Center, UNITED STATES

## Abstract

Tumor mutation load (TML) has been proposed as a biomarker of patient response to immunotherapy in several studies. TML is usually determined by tumor biopsy DNA (tDNA) whole exome sequencing (WES), therefore TML evaluation is limited by informative biopsy availability. Circulating cell free DNA (cfDNA) provided by liquid biopsy is a surrogate specimen to biopsy for molecular profiling. Nevertheless performing WES on DNA from plasma is technically challenging and the ability to determine tumor mutation load from liquid biopsies remains to be demonstrated. In the current study, WES was performed on cfDNA from 32 metastatic patients of various cancer types included into MOSCATO 01 (NCT01566019) and/or MATCHR (NCT02517892) molecular triage trials. Results from targeted gene sequencing (TGS) and WES performed on cfDNA were compared to results from tumor tissue biopsy. In cfDNA samples, WES mutation detection sensitivity was 92% compared to targeted sequencing (TGS). When comparing cfDNA-WES to tDNA-WES, mutation detection sensitivity was 53%, consistent with previously published prospective study comparing cfDNA-TGS to tDNA-TGS. For samples in which presence of tumor DNA was confirmed in cfDNA, tumor mutation load from liquid biopsy was correlated with tumor biopsy. Taken together, this study demonstrated that liquid biopsy may be applied to determine tumor mutation load. Qualification of liquid biopsy for interpretation is a crucial point to use cfDNA for mutational load estimation.

## Introduction

Tumor mutation load (TML) is reported as a valuable biomarker to predict response to immunotherapy. Several trials showed a correlation between patients harboring high TML and better responses to immunotherapy agents, in particular immune checkpoint inhibitors [1][2][3][4]. TML may also have prognostic value [5]. Evaluating TML in addition to mutation profile is of interest in the context of molecular triage trials, such as MOSCATO-01 (MOlecular Screening for CAncer Treatment Optimization) trial or MATCHR, two of the main molecular trials conducted at Gustave Roussy [6][7]. Indeed, reporting of TML for molecular board discussion is a valuable addition to orient patients towards immunotherapy trial, in complement to other molecular information mainly used for targeted therapy-based clinical trial orientation.

Currently the gold standard approach for the determination of mutation load is based on WES performed on tumor biopsy. Some authors also reported alternative approaches based on large target gene panel sequencing [8]. In the small fraction of cancer patients without suitable diagnostic tissue samples from surgical or endoscopic resections, this requires on-purpose tumor biopsy for molecular analysis. However, biopsy techniques present ethical and logistical challenges due to their invasiveness and risk of complications [9]. Moreover archive biopsy specimens do not always correspond to the molecular features of the disease at the time of treatment initiation. Even if tumor tissue biopsy remains the gold-standard specimen for diagnosis, liquid biopsies appear as an attractive alternative for tumor molecular assessment [10]. They are minimally invasive and circumvent the challenges related to on-purpose tumor biopsies; they can notably be repeated in a longitudinal manner. In the last few years, liquid biopsies have been increasingly explored for cancer molecular profiling and have shown potential great value for diagnosis, prognosis and theranostic applications. Indeed, longitudinal monitoring with liquid biopsies following therapy allows detecting the emergence of resistance mutations or any change in tumor molecular features. Therefore, it would be useful to evaluate TML from circulating cell-free DNA (cfDNA). Nevertheless performing WES on cfDNA is technically challenging, due to the low quantity of cfDNA that can be extracted from patient plasma, and given the low volumes of blood that may reasonably be dedicated to that analysis in a clinical context. Moreover, cfDNA is composed of small circulating DNA fragments shed into the plasma following apoptosis or necrosis of tumor cells through spontaneous release from cancer tissue, and possibly circulating tumor cells [10,11]. There are a few reports of attempts to analyze cfDNA-WES [12][13] [14][15]. Yet, TML from cfDNA was never investigated.

Our aim here was to assess and optimize WES from cfDNA, mainly for TML evaluation starting from clinical plasma samples collected from patients. TML derived from cfDNA-WES was compared to TML derived from tumor tissue DNA (tDNA), in order to evaluate whether cfDNA-based TML may be used as a surrogate for tDNA-based TML to select patient eligible for immunotherapy trials.

## Material and methods

### Patients and samples selection

**Fig 1:**
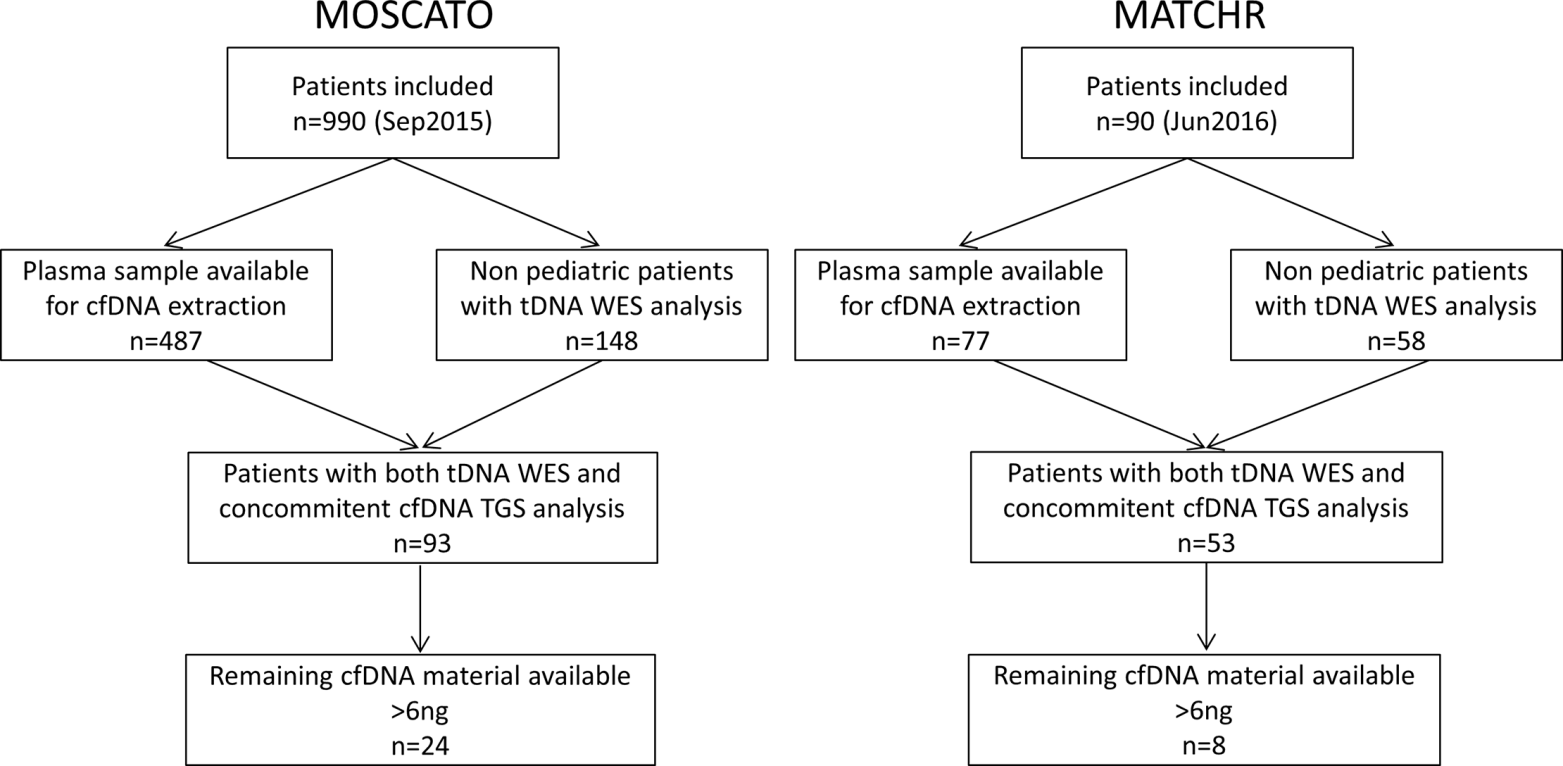
Study flow.

Written informed consent for genetic analysis of tumor and plasma samples was obtained for all patients at Gustave Roussy Cancer Center for the MOSCATO 01 and MATCHR protocols. These protocols were approved by local Institutional Review Boards CSET 2011/1755 (NCT01566019 trial) and CSET 2014/2144 (NCT02517892 trial), as well as regional ethics committee CPP ILE DE FRANCE VII, and were conducted according to the Good Clinical Practice Guidelines of the International Conference on Harmonization and the Declaration of Helsinki recommendations.We selected cfDNA samples from thirty-two non-consecutive patients harboring advanced cancer, based on availability of remaining cfDNA material [16], as well as availability of cfDNA-TGS and tDNA-WES results (Fig 1). Corresponding tumor biopsies presented a range of TML values. Cases of lung cancer were chosen preferentially since they present both low accessibility of tumor site and sensitivity to checkpoint inhibitors and therefore, determining TML from cfDNA-WES would be particularly valuable in this cancer type. In addition to 19 cases of lung cancers, samples from head and neck cancers (5 cases), prostate cancers (2 cases), cholangiocarcinoma (2 cases), colorectal cancers (2 cases), bladder cancer (1 case) and breast cancer (1 case) were selected. All patients had metastatic disease, except for patient P03, who had no known metastasis at the time of inclusion. Tumor biopsy cellularity mean and range for all samples was 52% [10–80] insuring fully contributive results for analysis performed with tDNA.

Based on cfDNA-TGS analysis, samples were classified as (1) circulating tumor DNA (ctDNA)-positive when known tumor mutations were detectable; (2) ctDNA-negative when known tumor mutations were not detectable in cfDNA; and (3) unknown when the sample did not have any known tumor mutation identified in the TGS panel. The terms “mutations” or “curated variants” are used here to designate variants, which were curated by a molecular geneticist [6] and may have a potential impact on disease understanding or management.

### Extraction of DNA

Tumor biopsies were obtained from either primary or metastatic sites and were immediately fresh-frozen as previously described [9,17]. Tumor cellularity was assessed by a senior pathologist on a haematoxylin and eosin slide from the same biopsy core as the one used for nucleic acid extraction as previously described [6]. Biopsy DNA was extracted using the AllPrep DNA/RNA Mini Kit (Qiagen) and quantified with Qubit 2.0 (ThermoFisher Scientific, Courtaboeuf, France).

For cfDNA extraction, blood samples were collected on the day before biopsy, except for patient P23 whose blood was collected 7 days before biopsy and patient P30 who had two biopsies, one on the day after blood draw and the other 172 days before blood draw. Blood samples (10 mL) were collected in EDTA-K2 tubes (BD Vacutainer, Beckton Dickinson and Company, Le Pont-de-Claix, France) and centrifuged for 10 minutes at 1,000 g within 4 hours of blood draw. The supernatant containing the plasma was further centrifuged at 14,000 g for 10 minutes at room temperature and stored at 80°C until analysis. DNA was extracted from 2 mL of plasma using QIAamp circulating nucleic acid kit (Qiagen), according to manufacturer's instructions, and suspended in 40 μL of AVE buffer. A real-time quantitative PCR TaqMan™ assay targeting GAPDH was used to measure plasma DNA concentration.

### Whole exome sequencing (tDNA and cfDNA)

Library preparation, exome capture, sequencing and data analysis were done by IntegraGen SA (Evry, France). Genomic DNA was captured using Agilent in-solution enrichment methodology (SureSelect SureSelect XT Clinical Research Exome, Agilent) with a biotinylated oligonucleotides probes library (SureSelect XT Clinical Research Exome—54 Mb, Agilent), followed by paired-end 75 bases massively parallel sequencing on Illumina HiSeq4000 [18]. Sequence capture, enrichment and elution were performed according to manufacturer’s instructions (SureSelect, Agilent) without modification except for library preparation performed with NEBNext® Ultra II kit (New England Biolabs®).

For cfDNA library preparation, an average of 25 ng and a minimum of 2 ng of cfDNA were engaged without an initial fragmentation. The profile was qualified on Fragment Analyzer. Only the peak around 166 base pairs was considered to quantify the extracted cfDNA. Paired-end adaptor oligonucleotides from the NEB kit were ligated on repaired A-tailed fragments, then SPRI purified with a ratio of 1.8X and enriched by 11 PCR cycles. The PCR was monitored with Evagreen on a Real-Time PCR System (StepOnePlus, ThermoFisher) and 2 cycles were added for samples which did not reach the PCR plateau.

For tDNA library preparation, 150 ng of each genomic DNA were fragmented by sonication and purified to yield fragments of 150–200 base pairs. Paired-end adaptor oligonucleotides from the NEB kit were ligated on repaired A-tailed fragments, then SPRI purified (ratio of 1.4X) and enriched by 8 PCR cycles. 1200 ng of these purified libraries were then hybridized to the SureSelect oligo probe capture library for 72 hr. After hybridization, washing and elution, the eluted fraction was PCR-amplified with 9 cycles, purified and quantified by QPCR to obtain sufficient DNA template for downstream applications. Each eluted-enriched DNA sample was then sequenced on an Illumina HiSeq4000 as paired-end 75 base pairs reads. Image analysis and base calling was performed using Illumina Real Time Analysis (2.7.3) with default parameters.

### WES bioinformatics analysis (tDNA and cfDNA)

Base calling was performed using the Real–Time Analysis software sequence pipeline (2) with default parameters. Sequence reads were mapped to the human genome build hg19 (GRCh37) using Elandv2e (Illumina, CASAVA1.8.2) allowing multiseeded and gapped alignments. Duplicated reads (e.g. paired–end reads in which the insert DNA showed identical start and end positions in the human genome) were removed.

CASAVA1.8.2 was used to call single–nucleotide variants (SNVs) and short insertions/deletions (maximum size was 300 nucleotides), taking into account all reads per position. Indels with Q(Indel) < 20, or regions with low mappability (QVCutoff < 90) were filtered out. An in-house algorithm was used to compare normal and tumor genotypes from exome sequencing data in order to determine the somatic nature of the variation. A somatic score was calculated for each variant ranging from 1 to 30, a score of 30 translating highest confidence index, taking into account frequencies and counts of mutated allele in both samples to minimize false positive variations. Finally, variants displaying mutated reads in the constitutional sample above 5% were considered as germline or false positive and eliminated to the somatic tab. The somatic variant caller handled indels similarly. Filtered variants were reviewed by an expert molecular medical geneticist to generate clinical reports and are considered as “curated variants” (also called “mutations”) in the current study.

CfDNA-WES sensitivity was defined as the ratio of the number of variants compared to the number of variants identified in the reference (either cfDNA-TGS or tDNA WES). CfDNA-WES pseudo-sensitivity was defined as the number of uncurated variants found in cfDNA-WES with a somatic score above 3 (corresponding to a sensitive detection) divided by the number of variants found in tDNA-WES with a somatic score above 5 (in order to consider as reference only high-confidence positions) and which positions were covered in cfDNA-WES, multiplied by 100. The term “pseudo” was used here because the number of variants was considered regardless of whether variants were matched between both WES analyses or not. Similarly, cfDNA-WES pseudo-specificity was defined as the number of uncurated variants found in tDNA-WES with a somatic score above 3 (in order to be sensitive) divided by the number of variants found in cfDNA-WES with a somatic score above 5 (in order to consider as reference only high-confidence positions) and which positions were covered in tDNA-WES, multiplied by 100.

### Tumor mutation load

Tumor mutation load was calculated by dividing the number of somatic mutations by the number of bases having a sequencing depth greater than 4. The somatic mutations used for tumor mutation load calculation were filtered as follows: somatic score > 4, Mutated Allele Frequency in tumor tissue ≥ 5%, mutated allele count in tumor tissue ≥ 3, Mutated Allele Frequency in constitutional tissue < 4%, IntegraGen heterozygous frequency ≤ 1%, IntegraGen homozygous frequency < = 1% and EVS & 100G & Exac variant frequency ≤ 0.5% and consequences on protein: stop, start, missense, splice for SNPs and inframe, frameshift for indels.

### Identification of somatic genomic mutations by TGS

Targeted sequencing (TGS) was performed as previously described [16]. Briefly, targeted sequencing libraries were generated using Ion AmpliSeq Library kit 2.0 according to manufacturer's instructions (Life Technologies). Plasma samples were analyzed with Cancer Hotspot Panel v2 (CHP2) targeting 50 cancer genes covered by 207 amplicons (Life Technologies). Tumor DNA samples were analyzed with a larger gene panel (74 genes) which included all the CHP2 amplicons. Primers used for amplification were partially digested by FuPa enzyme. Digested product was then ligated with adapters and barcodes, amplified and purified using Agencourt beads. Libraries were quantified using Qubit 2.0 Fluorometer. An equal amount of each library was pooled and amplified with Ion OneTouch 2 by emulsion PCR with Ion PGM Template OT2 200 Kit (Life Technologies). Sequencing was made using Ion Personal Genome Machine (PGM, Life Technologies). Sequencing data were analyzed with Torrent Suite Variant Caller 4.2 software and mapped to hg19. Variants were called if over 5 reads supported the variant and/or total base depth >50 and/or variant frequency >1% was observed. All variants identified were visually controlled on.bam files using Alamut v2.4.2 software (Interactive Biosoftware). All germline variants found in 1000 Genomes Project or ESP (Exome Sequencing Project database) with frequency >0.1% were removed. Filtered variants were reviewed, classified and annotated by an expert molecular medical geneticist according to available knowledge (COSMIC, TCGA and medical literature). Variants of interest were selected to generate clinical reports. All retained variants are reported here as “curated variants” or “mutations”. Analyses of tDNA and cfDNA were performed and interpreted independently.

## Results

### Feasability of cfDNA-WES

**Fig 2:**
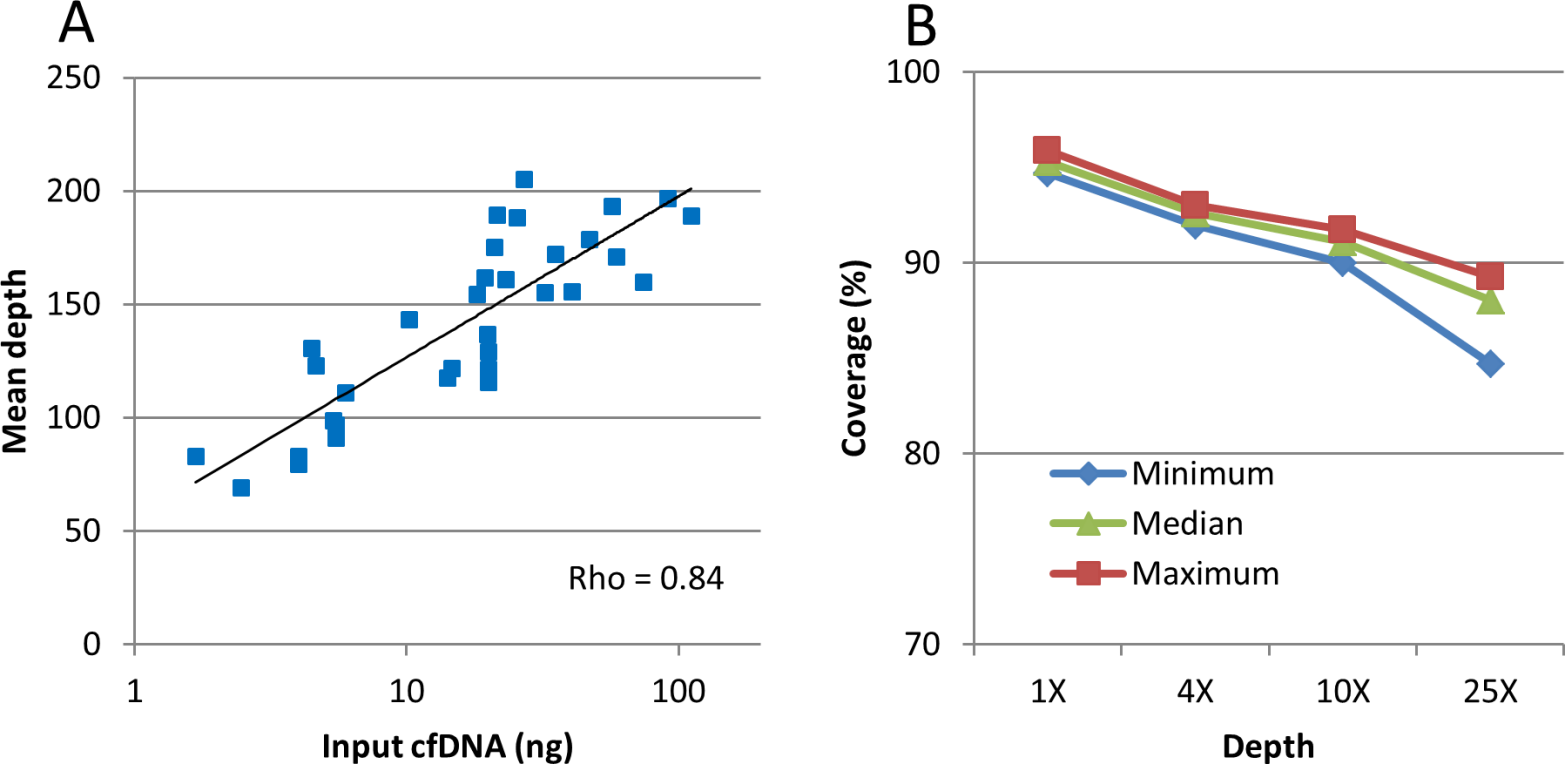
CfDNA-WES quality assessment. A. WES mean depth correlates logarithmically with input cfDNA quantity. B. High coverage at low depth for each sample.

The quantity of cfDNA used for WES depended on the amount of material extracted from plasma samples. Indeed, between 1 to 700 ng of cfDNA per milliliter of plasma are typically obtained, depending on various factors including tumor burden as we previously reported [16]. The sequencing protocol was set to be able to start from low cfDNA quantity, compatible with cfDNA quantity obtained in real life plasma samples. In our study, mean cfDNA input was 25 ng ± 13 [2–111]. Mean cfDNA-WES depth was 140X ± 20 [69–205], which was similar to WES sequencing depth typically used for tumor biopsy analysis in this clinical trial. Mean ten-x coverage was 91% ± 0.2 [90–91.7] and only 4.8% ± 0.1 [4.1% to 5.3%] of the target had no coverage, indicating a good quality of coverage. Mean depth increased logarithmically with DNA input, with an optimal depth observed around 20 ng of cfDNA input (Fig 2A and S1 Fig). The same bioinformatics pipeline was used as for tDNA and cfDNA. Indeed, a less stringent analysis of cfDNA was leading to an increased noise level and was therefore not used. Mean sequencing coverage was barely decreasing for low coverage region, indicating that all genomic region targeted by exome were present and sequenced, despite the fact that cfDNA is composed of small DNA fragments (Fig 2B).

### CfDNA-derived tumor mutation load

**Fig 3:**
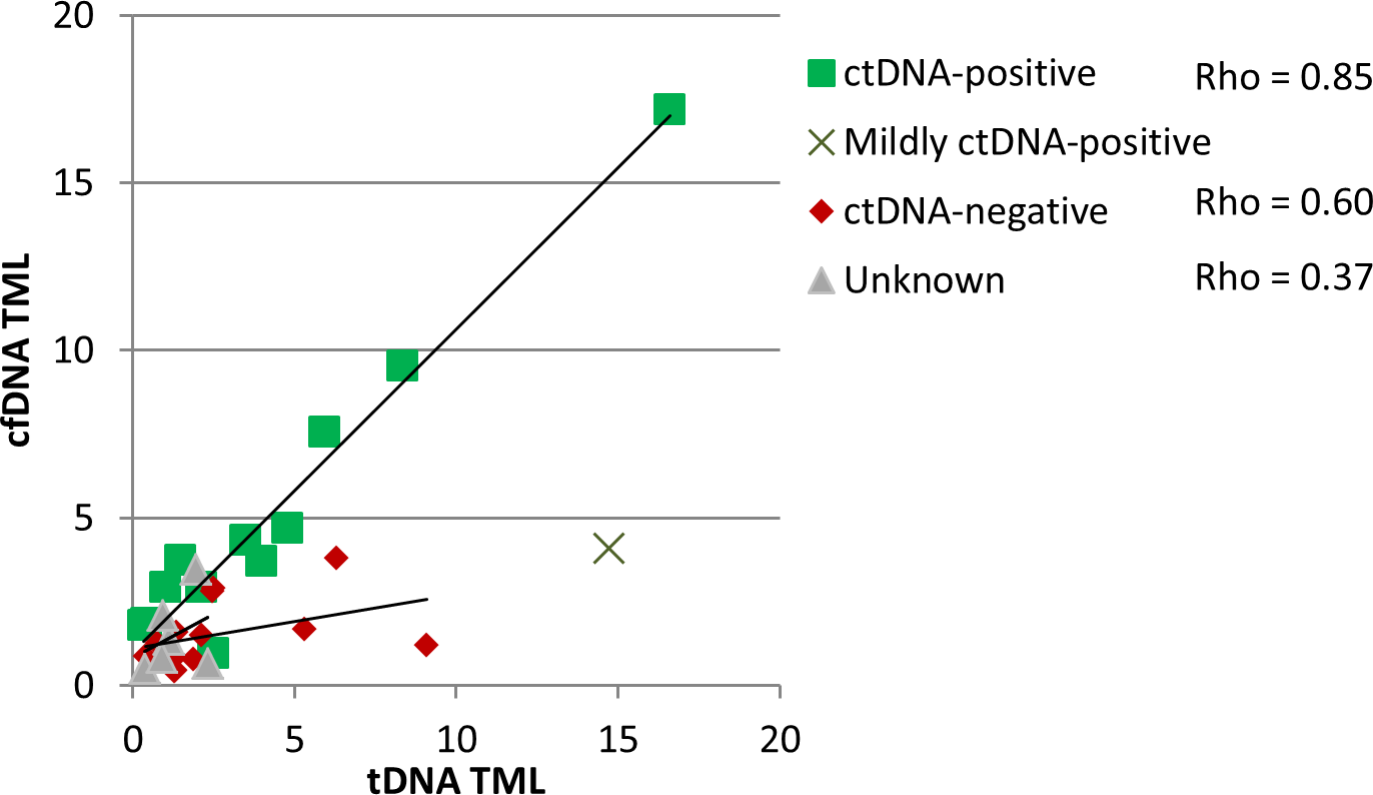
Tumor mutation load from cfDNA and tDNA are correlated in ctDNA-positive samples. Samples were classified as ctDNA-positive when at tDNA variants were also found in cfDNA-TGS with variant fraction in the same range, mildly ctDNA-positive when variant percentages was on average 5 times lower than in tDNA, ctDNA-negative when tDNA variants were not found in cfDNA and unknown when no tDNA variant was covered by the cfDNA-TGS panel. Pearson correlation coefficient of the linear regression for ctDNA-positive samples was 0.97. In a few cases, TML was observed to be higher in cfDNA than in tDNA. For example samples P07 (bladder cancer) harbored a 1.78variants per megabase TML in cfDNA compared to 0.34 variants per megabase TML in tDNA, which could be explained by low cellularity of biopsy (10%). The four cases with the strongest difference in TML between cfDNA and tDNA were P16, P17, P18 and P31. They all had a tumor mutation load lower than 5 in cfDNA and higher than 5 in tDNA.

Tumor mutation load was determined for tDNA and cfDNA-WES data (see S1 Table in supporting information). In tDNA, mean tumor mutation load was 3.4 variants per Mb ± 2 [0.3–16.6], corresponding to 162 variants ± 93 [16–786]. In plasma, mean tumor mutation load in cfDNA was 2.9 ± 1.7 variants per Mb [0.4–17.2], corresponding to 140 variants ±79 [19–818]. For most samples, tumor mutation loads were consistent between biopsy and plasma (Fig **3**).

For ctDNA-positive samples, cfDNA-derived TML was correlated with tDNA-derived TML (Spearman rank correlation was used, considering the low number of datapoints and the correlation coefficient rho was 0.85 for ctDNA-positive samples (Fig **3**)). All four cases with the strongest difference in TML between cfDNA and tDNA corresponded to ctDNA-negative (P16, P17 and P31) and mildly ctDNA-positive (P18) samples (with variant frequencies on average 5 times lower than in tDNA).

### Robustness of cfDNA-WES and comparison to tissue results

**Fig 4:**
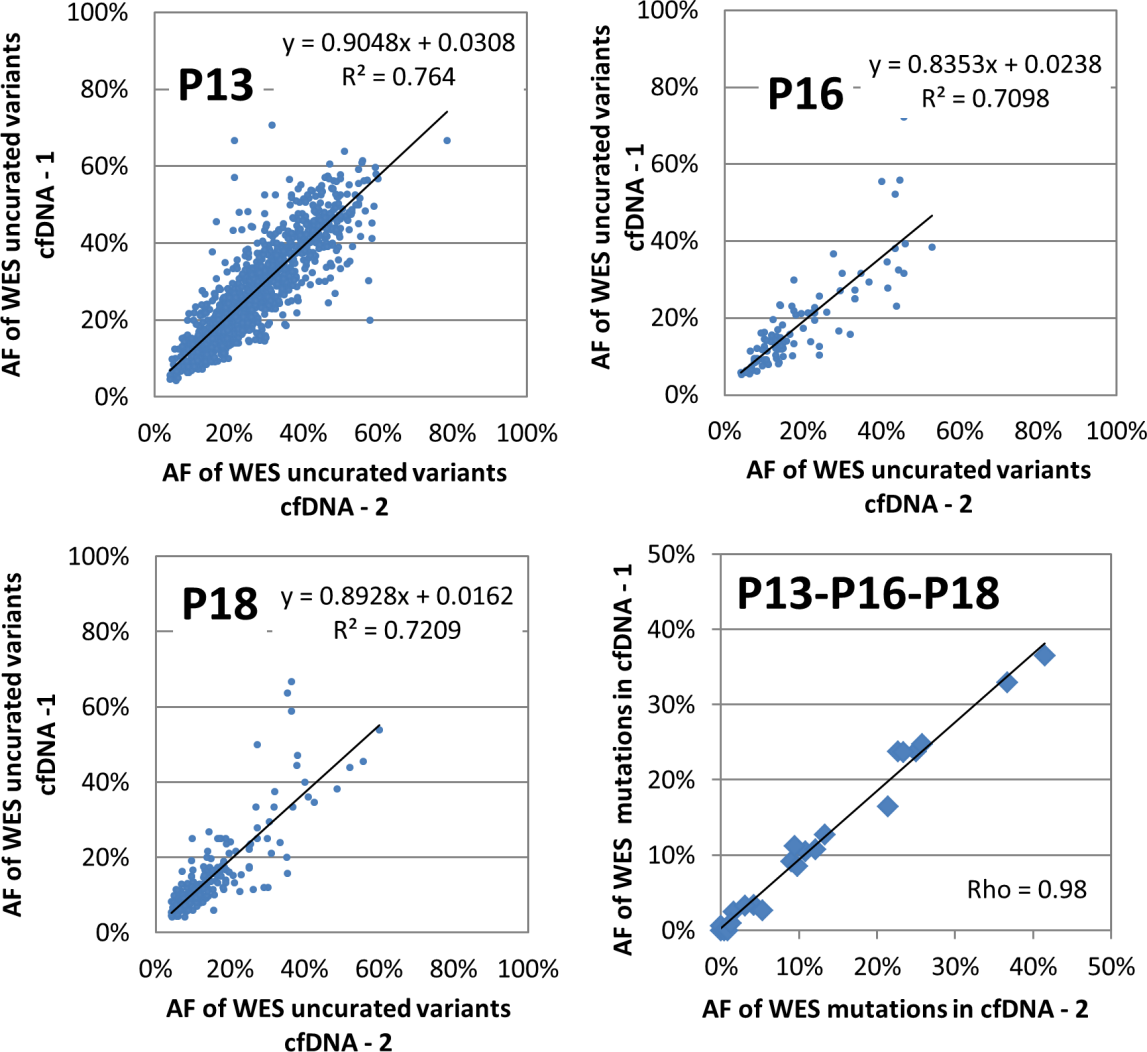
Correlation of variant percentages between duplicate whole exome sequencing of the same cfDNA extract. All somatic variants in common between each of the three duplicate samples are displayed. Lower right: Correlation of variant frequencies between duplicates for all curated variants (pooled results from patients P13, P16 and P18).

In order to investigate the robustness of cfDNA-WES, three samples were run twice independently starting from the same cfDNA extract but using different DNA input quantities. TML result was consistent between independent duplicates despite differences in DNA input quantities for samples P13 and P18. Only sample P16 presented low consistency, with only 10% of the uncurated tDNA-WES variants found in cfDNA-WES (Table 1). Yet in P16 tDNA-TGS, the only mutation identified which was covered by both panels was missing from cfDNA-TGS analysis, leading to the hypothesis that P16 cfDNA contained a low fraction of tumor circulating DNA. Variant frequencies of all uncurated variants present in the duplicate samples were reproducible (Fig 4).

**Table 1:** List of samples sequenced in duplicates by WES.

	cfDNA input (ng)	cfDNA mean depth	cfDNA tumor mutation load	tDNA tumor mutation load	cfDNA-WES pseudo-sensitivity	cfDNA-WES pseudo-specificity
P13—dup1	91	197	17.18	16.6	91.1	80.5
P13—dup2	20	127	15.16		90.3	91.6
P16—dup1	74	160	3.8	6.31	10.4	8.1
P16—dup2	20	123	0.61		11.8	25.2
P18—dup1	41	156	4.08	14.73	36.7	50.7
P18—dup2	20	120	3.82		35.3	52.7

### Sensitivity of cfDNA-WES and ctDNA-positive status

**Fig 5:**
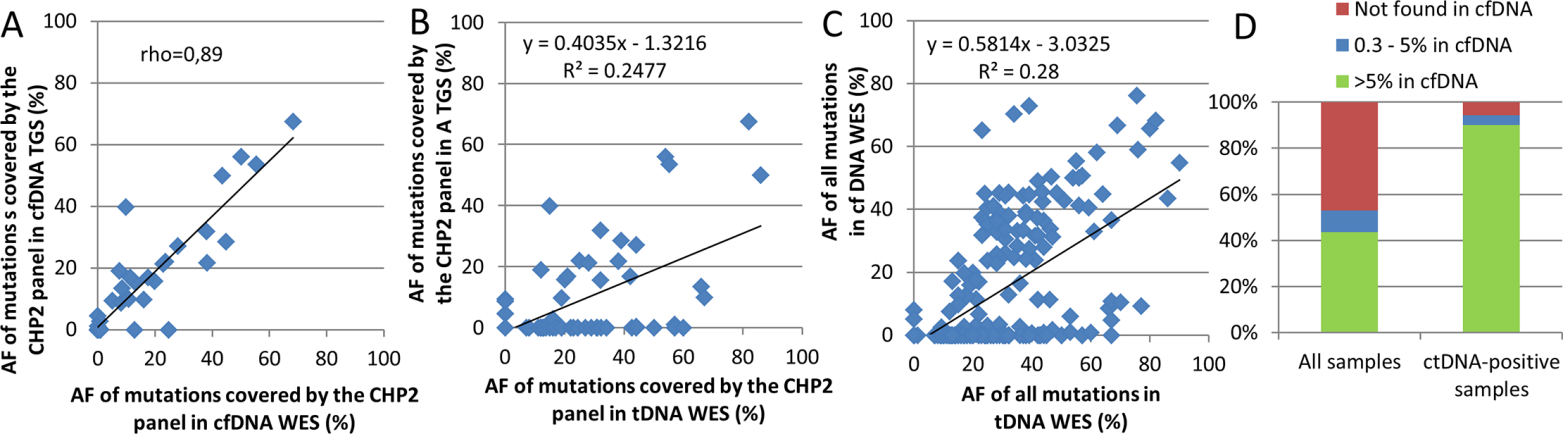
Allele frequencies of mutations. A and B. All mutations (curated variants) covered by the TGS panel. C. All mutations. D. Fraction of tDNA-WES variants found in cfDNA-WES (a total of 231 variants were found in tDNA-WES considering all samples, including 70 variants in ctDNA-positive samples).

WES results were then investigated at the level of individual variants. In cfDNA, amongst the 24 mutations found in TGS, 22 were also found in cfDNA-WES and variant frequencies were correlated (Spearman correlation coefficient 0.89 when considering all variants regardless of variant frequency, Fig 5A and S2 Table in supporting information). Therefore sensitivity of cfDNA-WES compared to cfDNA-TGS was 92%. In addition, 3 variants in cfDNA-WES with very low variant frequency (1%) were not detected in cfDNA-TGS.

Sensitivity of cfDNA-WES compared to tDNA-WES was also investigated. Amongst the 47 mutations covered by the TGS-panel identified in tDNA-WES, 24 were found in cfDNA-WES, therefore sensitivity of cfDNA-WES compared to tDNA-WES was 51% when focusing on areas covered by the TGS panel. When considering all 213 mutations found in tDNA-WES independently of the TGS-panel coverage, 113 were found in cfDNA-WES. Therefore, an overall sensitivity of 53% was observed for cfDNA-WES compared to tDNA-WES.

Thirteen ctDNA-positive samples (41%), 13 ctDNA-negative and 6 unknown status samples were identified. One ctDNA-positive sample was mildly-positive (4 variants found in cfDNA but with frequencies on average 5 times lower than in tDNA). When considering ctDNA-positive samples only, a total of 70 mutations were identified in tDNA WES independently of TGS-panel coverage, amongst which 66 were found in cfDNA-WES. Therefore, an overall sensitivity of 94% was obtained between cfDNA-WES and tDNA-WES in ctDNA-positive samples.

Two mutations were identified in cfDNA which were absent from both tDNA-WES and tDNA-TGS in 2 patients out of 32 (7%). The first was p.(Pro856Leu) in ERBB2 for patient P01 at frequencies 9% and 8% respectively in cfDNA-TGS and cfDNA-WES. The second was hotspot variant p.(Glu542Lys) in PIK3CA for patient P30 at frequencies 9% and 5% respectively in cfDNA-TGS and cfDNA-WES. Those 2 variants found in both WES and TGS cfDNA analysis were considered as true positive mutation rising from a subclone tumor cell not represented in biopsy sites.

Total number of all uncurated variants resulting from the bioinformatics pipeline in both cfDNA- and tDNA-WES analyses were compared. When considering all samples, cfDNA-WES pseudo-sensitivity and pseudo-specificity means were 43% [4–93] and 33% [6–80] respectively. When considering only the 12 ctDNA-positive samples, cfDNA-WES pseudo-sensitivity and pseudo-specificity mean values were 74% [46–93] and 52% [18–80] respectively.

### Comparison of cfDNA with two biopsy sites

**Fig 6:**
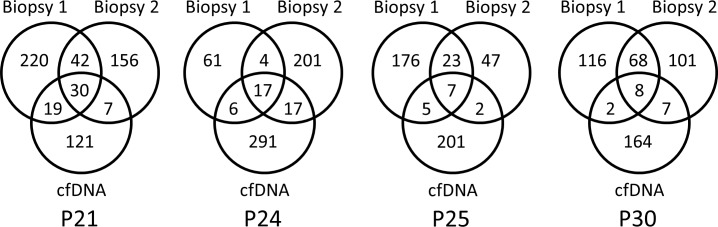
Number of variants in common between cfDNA and both corresponding biopsy sites. Liquid biopsies were collected on the day before the solid biopsies 1 and 2, except for patient P30, for whom biopsy 1 was collected 5 months before the liquid biopsy.

The added value of cfDNA-WES was evaluated for tumor heterogeneity assessment by comparing liquid biopsies to multiple matched tissue biopsy sites. WES analyses from two biopsy sites were available for four of the cfDNA samples (Fig 6). For 3 of them, both sites were synchronous with the plasma sample, whereas for P30, one biopsy was synchronous with the plasma sample, the other biopsy dated 5 months before. CfDNA-WES presented between 177 and 331 variants. Amongst them, between 9 and 49 were shared with a single biopsy site, while between 7 and 30 were shared with both biopsy sites. This data supports the hypothesis that liquid biopsy may capture the entire heterogeneity of the disease and enable tracking of subclonal dynamics [19].

## Discussion

Cell-free circulating DNA analysis constitutes an attractive approach to assess tumor mutational profile avoiding most of the caveats of tissue biopsies including risk of complications. Even if cfDNA analysis tends to become a gold standard for targetable driver mutation detection (especially for EGFR mutation in metastatic Non-Small Cell Lung Cancer), high throughput analysis such as whole exome sequencing have been poorly reported. Moreover, development of biomarkers for selection of patient for immunotherapy trials like Tumor Mutation Load, which is usually based on WES analysis, reinforce interest in performing such analysis starting from non-invasive samples. Therefore we investigate the possibility to enhance cfDNA-based analysis up to WES and TML evaluation. We also demonstrate our ability to perform WES with results equivalent to the few previously published reports. The latter were performed with various goals and the main studies are reported in Table 2. Sequencing quality data is equivalent to those obtained from tissue sequencing when starting from limited DNA quantity (1 to 100 ng). This is compatible with previous reports on patient plasma samples[12,14,15,20]. Moreover our study is, to our knowledge, the largest patient cohort published with cfDNA-WES analysis.

**Table 2:** Main publications reporting cfDNA-based whole exome sequencing.

Article	Cohort	cfDNA input	Depth and sensitivity achieved	Purpose/conclusion
Murtaza M et al. Non-invasive analysis of acquired resistance to cancer therapy by sequencing of plasma DNA. Nature. 2013; PMID: 23563269 [12]	2 breast, 3 ovarian and 1 NSCLC with 2 to 5 plasma samples/patient	Down to 2.3 ng	30x depth	CfDNA-WES is applicable to patient with high systemic tumor burden and enables disease monitoring.
Chan KC et al. Cancer genome scanning in plasma: detection of tumor-associated copy number aberrations, single-nucleotide variants, and tumoral heterogeneity by massively parallel sequencing. Clin Chem. 2013; PMID: 23065472 [21]	4 hepatocellular carcinoma, 1 breast/ovarian pre and post-operative plasma	1 to 5 μg	30x depth Sensitivity of 15 to 94% compared to WES biopsy	Shotgun MPS method is reported for genome view of SNV and CNV. Clinical and research putative implication need confirmation in larger cohort.
Klevebring D et al. Evaluation of exome sequencing to estimate tumor burden in plasma. PLoS One. 2014; PMID: 25133800 [15]	7 breast, 1 prostate plasma	1 to 10 ng	25-75x depth	cfDNA-WES is feasible but several inherent limitations need to be addressed.
Butler TM et al. Exome Sequencing of Cell-Free DNA from Metastatic Cancer Patients Identifies Clinically Actionable Mutations Distinct from Primary Disease. PLoS One. 2015; PMID: 26317216 [13]	1 sarcoma, 1 breast plasma	Over 100 ng	524X average depth	cfDNA-WES is feasible, but limited by low quantity of cfDNA. CfDNA variants are more correlated to metastasis than original tumor.
Murtaza M et al. Multifocal clonal evolution characterized using circulating tumour DNA in a case of metastatic breast cancer. Nat Commun. 2015; PMID: 26530965 [20]	9 plasma samples from 1 breast patient.	2 to 18 ng		CtDNA reflect clonal hierarchy. Truncal mutations are the best candidates to monitor tumor burden. Confirmation in larger cohort is needed.
Dietz S et al. Low Input Whole-Exome Sequencing to determine the Representation of the Tumor Exome in circulating DNA of Non-Small Cell Lung Cancer Patients. PLoS One. 2016; PMID: 27529345 [14]	NSCLC	10 ng	68x depth Pseudo-sensitivity 25–50% 57% specificity	Investigation of clinically relevant mutations and clonal heterogeneity.
Koeppel F et al. Current study.	32 patients (19 NSCLC, 5 HNSCC, 2 cholangio, 2 CRC, 2 prostate, 1 bladder, 1 breast)	2 to 110 ng	140x depth Sensitivity: 94% versus cfDNA-TGS 52% versus tDNA-WES	cfDNA-WES CML is feasible for patient with good ctDNA fraction

We demonstrate good repeatability of analysis through samples analysis performed twice, as well as good correlation between cfDNA-WES analysis and cfDNA-TGS analysis, underlying the confidence that we could have in the analysis. We also demonstrated the feasibility of TML determination based on cfDNA whole exome sequencing. Nevertheless performance of variant detection appears to be limited by the biological property of plasma samples, which does not necessary contain large enough amounts of ctDNA copies to obtain high sensitivity, as already reported by others [15]. Indeed, as expected, there was a weak correlation between variant frequencies of tDNA- and cfDNA-WES. Indeed, in contrast to a biopsy sample which is based on a cellular tissue structure, cfDNA contains of mix of tumor and normal DNA that do not reflect any cellular structure. The tumor DNA fraction in cfDNA potentially originates from multiple sites and/or from non-tumoral processes each bearing a specific allele frequency. Therefore, the term “allele frequency” is not fully appropriate to designate cfDNA variant frequency and cfDNA variant frequencies are not expected to correlate with the corresponding allele frequency in tumor site.

By definition, WES allows sequencing a much larger set of genes than TGS but depth of sequencing is typically much lower in WES than in TGS due to technical or cost considerations. The advantage of WES over TGS for oncology in a clinical context resides not so much in the identification of additional individual variants, but in global analysis (TML, mutation signature…) and cross-analysis with complementary techniques, such as RNAseq (for cross-validation of variants, detection of genes involved in both amplification and fusion transcripts, detection of monoallelic expression…). Indeed, despite the high number of individual variants identified in whole exome sequencing, only a limited set of variants correspond to potent targeted therapies and these may be screened by TGS on panels. Analysis of RNAseq from liquid biopsy has not yet been possible, due to a lack of technology to reliably isolate a sufficient quantity of mRNA from plasma or circulating exosomes. Therefore, so far, the application of cfDNA-WES for clinical oncology resides mainly in the determination of mutation load and potentially mutational profiles, or discovery of unexpected mutation not covered by standard gene panel, in particular when tumor biopsy is not available for various reasons or not contributive.

In addition, since sequential analysis may be performed much more easily with liquid biopsy than tissue biopsy, cfDNA-WES represents a powerful tool for translational research, to investigate the evolution of subclonal heterogeneity over time, in particular following various treatment options and tentatively identify new resistance mutations.

Our data supports the importance of evaluating ctDNA fraction amongst total plasma-derived cfDNA to identify the samples which are most suitable for cfDNA-WES analysis, in order to obtain informative analysis, especially tumor load calculation. Initial qualification of samples by targeted sequencing or any other method such as DNA methylation for example [22,23] appears to be a valuable approach to qualify cfDNA samples suitable for contributive WES analysis. For ctDNA-positive samples (i.e. when cfDNA contained tumor-originating DNA), sensitivity of cfDNA-WES compared to tDNA-WES was consistent with previous studies, considering primary sites and metastasis status of included patients [16]. cfDNA-WES enabled a comprehensive snapshot of alterations in all coding regions and allowed reliable determination of tumor mutation load, regardless of tumor type and cfDNA TML was correlated to tDNA TML. It is important to note that all cases with low TML in tDNA also had low TML in cfDNA, therefore no strongly overestimated TML (which could be considered as “false positive”) was observed in cfDNA. This means that no patient would have been inadequately evaluated. Despite the limited depth typically achieved with WES compared to TGS, cfDNA-WES demonstrated excellent sensitivity compared to cfDNA-TGS. In this context WES analysis could be a valuable technique for wide tumor samples analysis for care or in clinical trials when tumor mutation load, tumor mutations, whole exome copy number analysis or rare unexpected mutation screening is needed.

Moreover, data resulting from comparing cfDNA with tDNA from multiple biopsy sites supports the hypothesis that liquid biopsy may capture the entire heterogeneity of the disease and enable tracking of subclonal dynamics [19], demonstrating liquid biopsy’s utility in deciphering clonal heterogeneity.

To our knowledge, this is the first report investigating the use of liquid biopsy to determine tumor mutation load. This study suggests that liquid biopsy-derived tumor mutation load may be used as a surrogate marker for sensitivity to immune checkpoint blockers, in particular in cases when tumor biopsy is not accessible or not contributive. This could be investigated further in a prospective study as part of an immune checkpoint blockers clinical trial.

## Supporting information

S1 Table List of patients with tumor mutation loads and cfDNA secreting status (NSCLC: Non-small-cell lung carcinoma, CRC: Colorectal cancer, HNSCC: Head and neck squamous cell carcinoma). (DOCX)

S2 Table List of mutations covered by the TGS panel. (DOCX)

S1 Fig CfDNA WES library size reaches a plateau beyond 20 ng of DNA input. (TIF)
