# Supplementary material for: Whole exome sequencing for determination of tumor mutation load in liquid biopsy from advanced cancer patients
Source: PLoS One. 2017 Nov 21;12(11):e0188174. doi: 10.1371/journal.pone.0188174 (PMC5697854; doi:10.1371/journal.pone.0188174)
Supplement: S1 Table — (DOCX) [file pone.0188174.s001.docx]

|  |  | **TML (mut/Mb)** | | **Nb of variants used for TML determination** | | **Nb of mutations** | | **Nb of mutations covered by TGS pannel** | | **Nb of mutations** |  |
| --- | --- | --- | --- | --- | --- | --- | --- | --- | --- | --- | --- |
| **Patient ID** | **Primary site** | **tDNA WES** | **cfDNA WES** | **tDNA WES** | **cfDNA WES** | **tDNA WES** | **cfDNA WES** | **tDNA WES** | **cfDNA WES** | **cfDNA TGS** | **cfDNA secreting status** |
| P01 | Prostate | 8.36 | 9.53 | 393 | 455 | 19 | 16 | 1 | 1 | 1 | ctDNA-positive |
| P02 | Cholangiocarcinoma | 2.14 | 1.49 | 101 | 71 | 3 | 1 | 2 | 1 | 0 | ctDNA-negative |
| P03 | HNSCC | 2.5 | 2.88 | 118 | 137 | 6 | 2 | 1 | 0 | 0 | ctDNA-negative |
| P04 | NSCLC | 1.1 | 1.37 | 52 | 65 | 16 | 1 | 0 | 0 | 0 | Unknown |
| P05 | Breast | 1.95 | 3.45 | 92 | 164 | 18 | 18 | 0 | 0 | 0 | Unknown |
| P06 | Prostate | 1.02 | 2.91 | 48 | 139 | 2 | 2 | 1 | 1 | 1 | ctDNA-positive |
| P07 | Bladder | 0.34 | 1.78 | 16 | 85 | 5 | 5 | 1 | 1 | 1 | ctDNA-positive |
| P08 | NSCLC | 5.92 | 7.56 | 280 | 360 | 5 | 5 | 1 | 1 | 1 | ctDNA-positive |
| P09 | Cholangiocarcinoma | 0.76 | 1.26 | 36 | 60 | 2 | 1 | 1 | 0 | 0 | ctDNA-negative |
| P10 | CRC | 2.13 | 2.91 | 101 | 139 | 7 | 7 | 5 | 5 | 5 | ctDNA-positive |
| P11 | CRC | 3.5 | 4.32 | 165 | 206 | 7 | 7 | 4 | 4 | 4 | ctDNA-positive |
| P12 | HNSCC | 4 | 3.71 | 190 | 177 | 4 | 3 | 1 | 1 | 1 | ctDNA-positive |
| P13 | NSCLC | 16.6 | 17.18 | 786 | 818 | 8 | 8 | 0 | 0 | 0 | ctDNA-positive |
| P14 | NSCLC | 2.31 | 0.65 | 109 | 31 | 7 | 1 | 0 | 0 | 0 | Unknown |
| P15 | HNSCC | 0.91 | 2.08 | 43 | 99 | 1 | 1 | 0 | 0 | 0 | Unknown |
| P16 | NSCLC | 6.31 | 3.8 | 299 | 180 | 6 | 1 | 1 | 0 | 1 | ctDNA-negative |
| P17 | HNSCC | 5.31 | 1.67 | 252 | 80 | 7 | 3 | 1 | 1 | 0 | ctDNA-negative |
| P18 | NSCLC | 14.73 | 4.08 | 695 | 194 | 15 | 11 | 3 | 3 | 4 | Mildly ctDNA-positive |
| P19 | HNSCC | 1.48 | 3.73 | 70 | 177 | 7 | 5 | 1 | 1 | 1 | ctDNA-positive |
| P20 | NSCLC | 2.47 | 2.79 | 117 | 133 | 7 | 1 | 3 | 0 | 0 | ctDNA-negative |
| P21 | NSCLC | 2.5 | 0.95 | 119 | 45 | 3 | 2 | 2 | 2 | 2 | ctDNA-positive |
| P22 | NSCLC | 1.31 | 0.44 | 62 | 21 | 4 | 1 | 3 | 0 | 0 | ctDNA-negative |
| P23 | NSCLC | 1.29 | 0.4 | 61 | 19 | 6 | 2 | 2 | 1 | 0 | ctDNA-negative |
| P24 | NSCLC | 1.87 | 0.76 | 89 | 36 | 14 | 2 | 1 | 0 | 0 | ctDNA-negative |
| P25 | NSCLC | 1.29 | 0.8 | 61 | 38 | 6 | 1 | 3 | 0 | 0 | ctDNA-negative |
| P26 | NSCLC | 0.4 | 0.86 | 19 | 41 | 3 |  | 2 | 0 | 0 | ctDNA-negative |
| P27 | NSCLC | 0.38 | 0.51 | 18 | 24 | 3 | 2 | 0 | 0 | 0 | Unknown |
| P28 | NSCLC | 0.88 | 0.82 | 42 | 39 | 1 | 0 | 0 | 0 | 0 | Unknown |
| P29 | NSCLC | 1.36 | 1.58 | 65 | 75 | 6 | 0 | 5 | 0 | 0 | ctDNA-negative |
| P30 | NSCLC | 0.42 | 1.84 | 20 | 87 | 2 | 2 | 0 | 1 | 1 | ctDNA-positive |
| P31 | NSCLC | 9.08 | 1.2 | 436 | 57 | 12 | 1 | 1 | 0 | 0 | ctDNA-negative |
| P32 | NSCLC | 4.8 | 4.69 | 229 | 223 | 1 | 1 | 1 | 1 | 1 | ctDNA-positive |
