# Supplementary material for: Whole exome sequencing for determination of tumor mutation load in liquid biopsy from advanced cancer patients
Source: PLoS One. 2017 Nov 21;12(11):e0188174. doi: 10.1371/journal.pone.0188174 (PMC5697854; doi:10.1371/journal.pone.0188174)
Supplement: S2 Table — (DOCX) [file pone.0188174.s002.docx]

| **Patient ID** | **Gene** | **AminoAcid Change** | **hg19 mutation description** | **Mutation frequency** | | | | |
| --- | --- | --- | --- | --- | --- | --- | --- | --- |
|  |  |  |  | **tDNA TGS (%)** | **tDNA WES (%)** | **cfDNA TGS (%)** | **cfDNA WES (%)** | **cfDNA WES depth** |
| P01 | ERBB2 | p.(Pro856Leu) | chr17:g.37881375C>T | 0 | 0 | 9 | 8 | 357 |
| P01 | TP53 | p.(Gln100*) | chr17:g.7579389G>A | 38 | 50 | 0 | 0 | 217 |
| P02 | SMAD4 | p.(Arg445*) | chr18:g.48603032C>T | 9 | 5 | 0 | 0 | 279 |
| P02 | IDH1 | p.(Arg132Cys) | chr2:g.209113113G>A | 27 | 19 | 0 | 1 | 273 |
| P03 | TP53 | p.(Leu35Phe) | chr17:g.7579582C>A | 52 | 43 | 0 | 0 | 69 |
| P06 | TP53 | p.(Val216Met) | chr17:g.7578203C>T | 80 | 82 | 68 | 68 | 101 |
| P07 | FGFR3 | p.(Ser249Cys) | chr4:g.1803568C>G | 25 | 12 | 19 | 8 | 106 |
| P08 | TP53 | p.(Tyr236Cys) | chr17:g.7577574T>C | 91 | 86 | 50 | 43 | 214 |
| P09 | PTEN | p.(Leu265fs) | chr10:g.89717770delA | 21 | 22 | 0 | 0 | 208 |
| P10 | APC | p.(Ser1465Trpfs*3) | chr5:g.112175676_112175677delAG | 24 | 20 | 16 | 20 | 132 |
| P10 | APC | p.(Arg1114*) | chr5:g.112174631C>T | 24 | 25 | 22 | 24 | 330 |
| P10 | BRAF | p.(Val600Glu) | chr7:g.140453136A>T | 31 | 32 | 32 | 38 | 310 |
| P10 | FBXW7 | p.(Arg505Cys) | chr4:g.153247289G>A | 39 | 38 | 22 | 38 | 461 |
| P10 | TP53 | p.(Arg282Trp) | chr17:g.7577094G>A | 51 | 54 | 56 | 50 | 403 |
| P11 | APC | p.(Thr1493Argfs*14) | chr5:g.112175766delC | 12 | 19 | 10 | 16 | 223 |
| P11 | APC | p.(Arg876*) | chr5:g.112173917C>T | 11 | 21 | 17 | 17 | 286 |
| P11 | KRAS | p.(Gly12Asp) | chr12:g.25398284C>T | 13 | 28 | 21 | 23 | 202 |
| P11 | TP53 | p.(Arg273Ser) | chr17:g.7577121G>T | 25 | 44 | 27 | 28 | 322 |
| P12 | TP53 | p.(Arg273His) | chr17:g.7577120C>T | 32 | 39 | 29 | 45 | 286 |
| P16 | KRAS | p.(Gly13Asp) | chr12:g.25398281C>T | 61 | 57 | 1 | 0 | 163 |
| P17 | PTEN | p.(Arg130*) | chr10:g.89692904C>T | 45 | 44 | 0 | 1 | 260 |
| P18 | APC | p.(Asp1498Asn) | chr5:g.112175783G>A | 13 | 0 | 4 | 0 | 246 |
| P18 | CTNNB1 | p.(Ser37Cys) | chr3:g.41266113C>G | 18 | 16 | 3 | 1 | 157 |
| P18 | STK11 | p.(Ile177Met) | chr19:g.1220438C>G | 65 | 66 | 13 | 9 | 199 |
| P18 | TP53 | p.(Val173Gly) | chr17:g.7578412A>C | 61 | 67 | 10 | 11 | 296 |
| P19 | TP53 | p.(Arg283Glnfs*29) | chr17:g.7577090_7577091ins AGATTCTCTTCCTCTGTGC | 39 | 15 | 40 | 10 | 209 |
| P20 | EGFR | p.(Thr790Met) | chr7:g.55249071C>T | 18 | 17 | 0 | 0 | 191 |
| P20 | EGFR | p.(Leu858Arg) | chr7:g.55259515T>G | 37 | 29 | 0 | 0 | 162 |
| P20 | TP53 | p.(Glu224*) | chr17:g.7578179C>A | 49 | 57 | 0 | 0 | 105 |
| P21 | EGFR | p.(Glu746_Ser752delinsVal) | chr7:g.55242466_55242479delGAATTAAGAGAAGinsC | 45 | 32 | 16 | 13 | 82 |
| P21 | TP53 | p.(Cys135Serfs*35) | chr17:g.7578526delC | 57 | 42 | 17 | 11 | 68 |
| P22 | IDH1 | p.(Arg132Cys) | chr2:g.209113113G>A | 22 | 24 | 0 | 0 | 191 |
| P22 | TP53 | p.(His179Arg) | chr17:g.7578394T>C | 26 | 31 | 0 | 0 | 233 |
| P22 | BRAF | p.(Val600Glu) | chr7:g.140453136A>T | 22 | 31 | 0 | 0 | 160 |
| P23 | TP53 | p.(His214Arg) | chr17:g.7578208T>C | 9 | 7 | 0 | 0 | 125 |
| P23 | EGFR | p.(Leu861Arg) | chr7:g.55259524T>G | 53 | 60 | 0 | 1 | 125 |
| P24 | CTNNB1 | p.(Ser37Phe) | chr3:g.41266113C>T | 23 | 8 | 0 | 0 | 98 |
| P25 | EGFR | p.(Thr790Met) | chr7:g.55249071C>T | 17 | 12 | 0 | 0 | 149 |
| P25 | EGFR | p.(Lys745_Glu749del) | chr7:g.55242465_55242477delAGGAATTAAGAG | 26 | 16 | 0 | 0 | 101 |
| P25 | PTEN | p.(Gln132Asp) | chr10:g.89692911G>A | 25 | 27 | 0 | 0 | 151 |
| P26 | EGFR | p.(Glu746_Ser752delinsVal) | chr7:g.55242467_55242486delAATTAAGAGAAGCAACATCinsT | 10 | 11 | 0 | 0 | 95 |
| P26 | TP53 | p.(Cys238Phe) | chr17:g.7577568C>A | 13 | 13 | 0 | 0 | 75 |
| P29 | EGFR | p.(Cys797Ser) | chr7:g.55249091T>A | 14 | 12 | 0 | 0 | 142 |
| P29 | TP53 | p.(Arg282Trp) | chr17:g.7577094G>A | 13 | 13 | 0 | 0 | 279 |
| P29 | CTNNB1 | p.(Gly34Arg) | chr3:g.41266103G>A | 6 | 14 | 0 | 0 | 128 |
| P29 | EGFR | p.(Thr790Met) | chr7:g.55249071C>T | 31 | 23 | 0 | 0 | 172 |
| P29 | EGFR | p.(Glu746_Glu749del) | chr7:g.55242465_55242477delAGGAATTAAGAGinsA | 47 | 32 | 0 | 0 | 131 |
| P30 | PIK3CA | p.(Glu542Lys) | chr3:g.178936082G>A | 0 | 0 | 9 | 5 | 138 |
| P31 | TP53 | p.(Ala159Pro) | chr17:g.7578455C>G | 19 | 13 | 0 | 0 | 134 |
| P32 | TP53 | p.(Cys229*) | chr17:g.7577594A>T | 61 | 55 | 54 | 55 | 159 |
