# Supplementary figures and images for: Whole exome sequencing for determination of tumor mutation load in liquid biopsy from advanced cancer patients
Source: PLoS One. 2017 Nov 21;12(11):e0188174. doi: 10.1371/journal.pone.0188174 (PMC5697854; doi:10.1371/journal.pone.0188174)

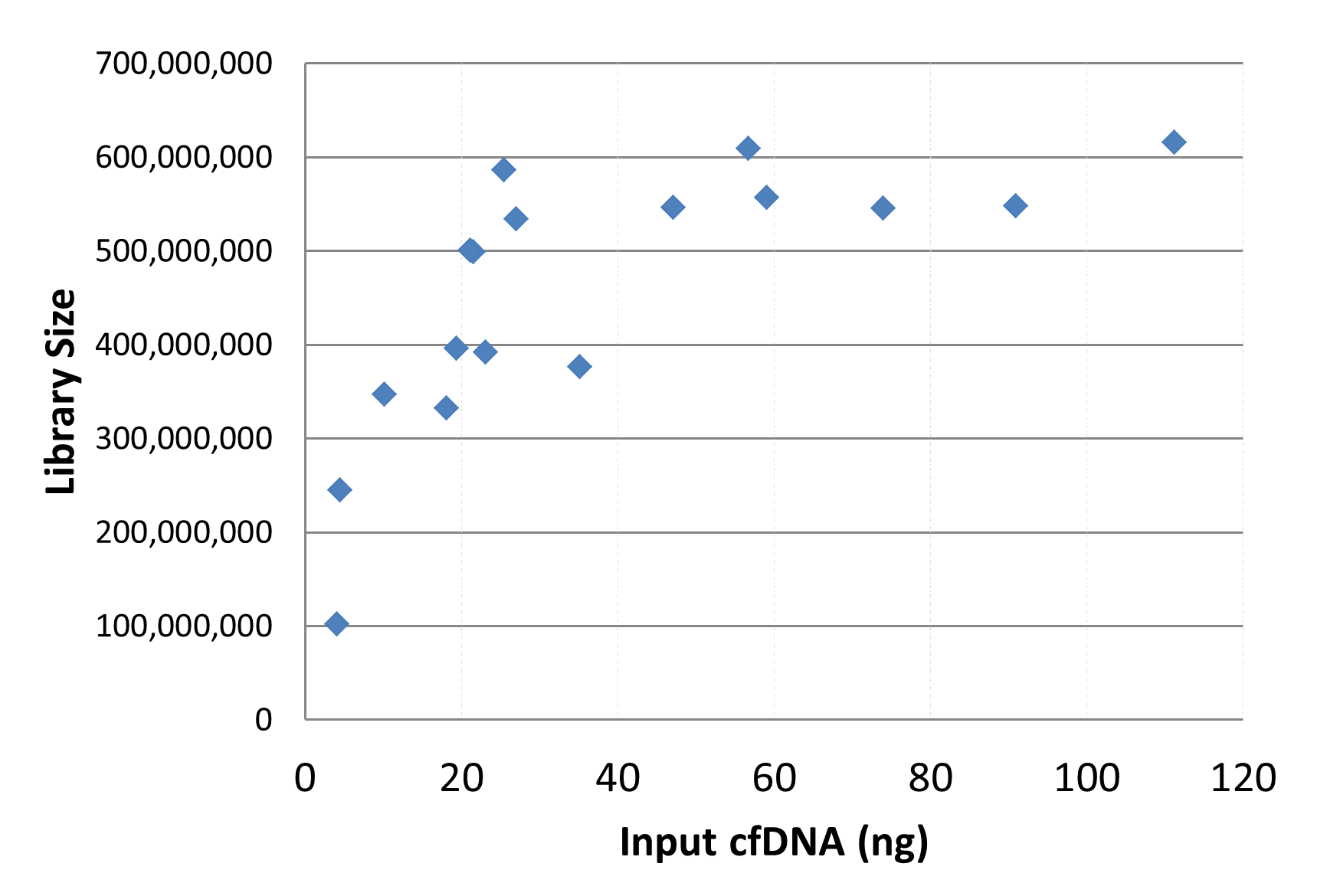

Supplement: S1 Fig — (TIF) [file pone.0188174.s003.TIF]
